# Study on the Low-Velocity Impact Response and Damage Mechanisms of Thermoplastic Composites

**DOI:** 10.3390/polym16060791

**Published:** 2024-03-13

**Authors:** Liu Han, Hui Qi, Jinshui Yang, Fuqing Chu, Changliang Lin, Pingan Liu, Qian Zhang

**Affiliations:** 1 College of Aerospace and Civil Engineering, Harbin Engineering University, Harbin 150001, China; hanl019@avic.com (L.H.); yangjinshui@hrbeu.edu.cn (J.Y.); coungcg@hrbeu.edu.cn (F.C.); liupingan@hrbeu.edu.cn (P.L.); 2 AVIC Harbin Aircraft Industry Croup Co., Ltd., Harbin 150066, China; linzl001@avic.com; 3 Qingdao Innovation and Development Base, Harbin Engineering University, Qingdao 266000, China; 4 School of Mechanical Engineering, Hefei University of Technology, Hefei 230009, China; zq_hfut@hfut.edu.cn

**Keywords:** thermoplastic composites, low-velocity impact, energy absorption characteristics, simulation

## Abstract

A comparative experimental and numerical study of the impact behaviour of carbon-fiber-reinforced thermoplastic (TP) and thermoset (TS) composites has been carried out. On the one hand, low velocity impact (LVI) tests were performed on TP and TS composites with different lay-up sequences at different energy levels, and the damage modes and microscopic damage mechanisms after impact were investigated using macroscale inspection, C-scan inspection, and X-ray-computed tomography. The comparative results show that the initial damage valve force under LVI depends not only on the material, but also on the layup sequence. The initial valve force of the P2 soft layer with lower stiffness is about 11% lower than that of the P1 quasi-isotropic layer under the same material, while the initial valve force of thermoplastic composites is about 28% lower than that of thermoset composites under the same stacking order. Under the same stacking order and impact energy level, the damage area and depth of TP composites are smaller than those of TS composites; while under the same material and impact energy level, the indentation depth of P2 plies is greater than that of P1 plies, and the damage area of P2 plies is smaller than that of P1 plies, but the change of thermoplastic composites is not as obvious as that of thermoset composites. This indicates that TP composites have a higher initial damage threshold energy and impact resistance at the same lay-up order, while increasing the lay-up ratio of the same material by 45° improves the impact resistance of the structure. In addition, a damage model based on continuum damage mechanics (CDM) was developed to predict different damage modes of thermoplastic composites during low velocity impact, and the analytical results were compared with the experimental results. At an impact energy of 4.45 J/mm, the error of the initial damage valve force is 5.26% and the error of the maximum impact force is 4.36%. The simulated impact energy and impact velocity curves agree with the experimental results, indicating that the finite element model has good reliability.

## 1. Introduction

Composite laminates are widely used in aerospace, automotive, shipbuilding, and other fields due to their high strength, high stiffness, and better fatigue resistance [1,2,3,4,5]. However, composite materials are very sensitive to low-velocity impacts; under a low-velocity impact, composite materials will produce large internal delamination (delamination, matrix cracking, fiber bundle separation, fiber breakage, etc.), while only leaving a small indentation on the impact surface. This invisible damage to the surface can significantly reduce the performance of the material, even reducing the structural load-bearing capacity to half of what it was originally intended to be [1,2,3]. Therefore, low-velocity impact has become a serious threat to the safe use of composite materials [6,7,8,9]. Improving the impact resistance of composites has become a hotspot for many researchers, and some experts improve the impact resistance of composites by changing the fabric structure and toughening the resin, while others shift the research direction to thermoplastic composites [10]. Research on the impact properties and fracture toughness of thermoplastic composites has shown that TP composites have higher delamination resistance because the higher matrix toughness can retard crack propagation [11]. In addition, TP composites are fast to manufacture, have low moisture absorption, are weldable, are recyclable, and have unlimited shelf life and good stability in extreme environments [10,11,12,13,14].

At present, many experts have investigated the low-velocity impact performance of thermoset composites with different impact energies, ply thicknesses, and stacking sequences by means of multiple low-velocity impact tests and borrowing different testing equipment to analyze the damage mechanism of thermoset composites under low secular impact [15,16,17,18,19]. Léonard et al. [16] detected the low-velocity impact damage of carbon-fiber-reinforced plastic (CFRP) laminates by means of X-ray computed tomography (CT), and extracted the thickness distribution of the damage of the laminates. Lu et al. [17] analyzed the damage of two specimens with the same geometry and lay-up after impact by XRM and ultrasonic C-scan. Gohel et al [18] investigated the characteristics of different TP composites under low velocity impact at different energy levels. Vieille et al. [20] used ultrasonic C-scanning and microscopy to compare the LVI response characteristics and damage mechanisms of composites with three different resins (epoxy, polyphenylene sulphide, and poly(ether ether ketone) (PEEK)) and found that PEEK composites showed better impact resistance among all three. Garcea et al. [21,22] performed incremental in situ fatigue loading of toughened epoxy composites, and used synchrotron X-ray-computed tomography to observe the effect of toughened particles on microcrack growth. Zhibin ZHAO et al. [23] conducted a study on glass-fiber-reinforced thermoplastic polypropylene and carbon-fiber-reinforced thermoset epoxy laminates regarding low velocity impact behaviour, their infrared radiation characteristics were investigated, and techniques such as C-scan and active/passive thermography were used to analyze the damage evolution and damage patterns of the laminates. Although most of them have analyzed the damage characteristics of thermoset or thermoplastic composites under low-velocity impact using different testing devices, there are fewer studies comparing the low-velocity impact damage of thermoplastic composites and thermoset composites; in particular, there is a lack of microscopic analyses of the effects of the plastic deformation capacity of the matrix on the impact performance of thermoplastic and thermoset composites.

In numerical analyses, small-scale upscaling models have been used to simulate the damage pattern and damage extension of composites under low-speed impacts, which can lead to “softening” of the material. Robin Olsson et al. [24] developed a computational model to analyze the damage generation and extension during the static response caused by a heavy punch, and analyzed the impact damage under different stacking sequences, geometries, and boundary conditions, while Lee et al. [25] developed a computational model based on the Rayleigh-Ritz method [26] and the theory of wide-plate deflection. The model assumes that the deformation pattern under a low-velocity impact is consistent with quasi-static loading, and the model establishes a method for assessing the residual compressive strength after impact based on fracture energy according to the model’s prediction of the damage zone. With the development of finite element technology, plasticity theory, fracture mechanics, and failure criterion are continuously introduced into the finite element, which can successfully predict different composite material damage modes, such as matrix cracking, interlaminar delamination, fiber breakage and other damage modes. Moreover, nonlinear shear and damage-induced irreversible strain of composites can be assumed, and these damage-modelling patterns can be implemented in the user material subroutine VUMAT of the ABAQUS/explicit finite element program [27]. Singh et al. [28] proposed a three-dimensional elasto-plastic damage model for fiber-reinforced plastic composites to simulate progressive damage and damage-induced inelastic deformation under low velocity impacts. Schwab et al. [29] simulated the mechanical behaviour of a large deformable body hitting a composite component using the finite element method. Romano et al. [30] numerically analyzed the impact damage based on the stiffness degradation method, and experimentally verified the numerical model for progressive damage analysis. However, the most typical property difference between thermoplastic composites and thermoset composites is that the resin has good toughness and a certain nonlinear behaviour during loading, which is more obvious for shear strain.

More studies have been carried out on low-velocity impact on thermoset composites, including on the effects of layer thickness, stacking sequence, and curing process parameters on the impact behaviour under low-velocity impacts [31,32,33]. For thermoplastic composites, the main focus has been on the observation of different damage modes generated under impact loading [34,35,36,37,38,39], such as fiber breakage, matrix cracking, and/or interlaminar delamination. Few works have comparatively analyzed the effects of low-velocity impacts on the damage modes of thermoplastic and thermoset composites on a macroscopic and microscopic basis, respectively. In this paper, the impact behaviour of carbon-fiber-reinforced thermoplastic (TP) and thermoset (TS) composites is investigated experimentally and numerically in a comparative manner. On the one hand, low velocity impact (LVI) tests were performed on TP and TS composites with different lay-up sequences at different energy levels, and the damage modes and microscopic damage mechanisms induced by LVI were analyzed using a variety of inspection methods such as macroscopic scale inspection, C-scan inspection, X-ray-computed tomography (XRCT), and scanning electron microscopy (SEM) SEM. In addition, a damage model based on continuous damage mechanics (CDM) is established to introduce shear nonlinearity for predicting various damage modes of thermoplastic composites during low-velocity impacts, and the analytical results are in good agreement with the experimental results in terms of impact force, energy absorption, and delamination damage modes, indicating that the damage model has good reliability.

## 2. Experiments

### 2.1. Materials

The carbon-fiber-reinforced TP composite used in the specimens is AS4D/PEEK polymer prepreg provided by Barrday Company (Charlotte, NC, USA), the matrix material is TP resin PEEK, and the reinforcement is AS4D carbon fiber with 12 K. The carbon-fiber-reinforced TS composites used are the CCF300/Epoxy polymer. Table 1 gives the properties of the AS4D and CCF300 carbon fibers; it is shown that these two fibers have similar characteristics. Table 2 gives the properties of the PEEK and CCF300 matrix. Table 3 shows the material properties of the AS4D/PEEK TP composite and CCF300/Epoxy.

### 2.2. Method

**Figure 1:**
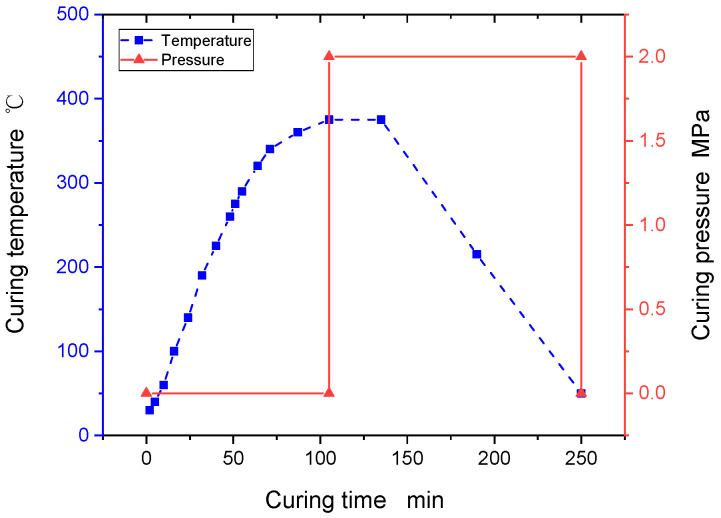
Hot press process curve for thermoplastic composites.

Thermoplastic composite laminates are manufactured in four steps using the hot compression moulding process. In the first step, the prepreg is cut to the size of the large sheet, and the cut prepreg is laid out according to the design angle of the lay-up and spot welded layer by layer. Before moulding, a mould release agent should be used to treat the mould to avoid difficulty in mould release. In the second step, after the film is closed, a pre-pressure of 0.01 MPa is first applied to expel the gas between the layers, and the hot press is divided into three processes to increase the temperature to 385 °C. Then, the pressure is increased to 1.2 MPa, and the insulation is kept at a constant pressure for 30 min to make the resin and fibers form a good adhesion with each other (see Figure 1 for the curve of the hot pressing process). The third step is to cool the large plate to room temperature at a cooling rate of 3 °C/min, and keep the pressure constant during the cooling process. The fourth step is to cut the board into test pieces according to size, and a test indicator is used during the cutting process to ensure that the accumulation of fiber direction error does not exceed 2°. In addition, a water-cooled diamond tool is used to cut the test pieces to avoid overheating and carbonization of the substrate during the cutting process.

**Figure 2:**
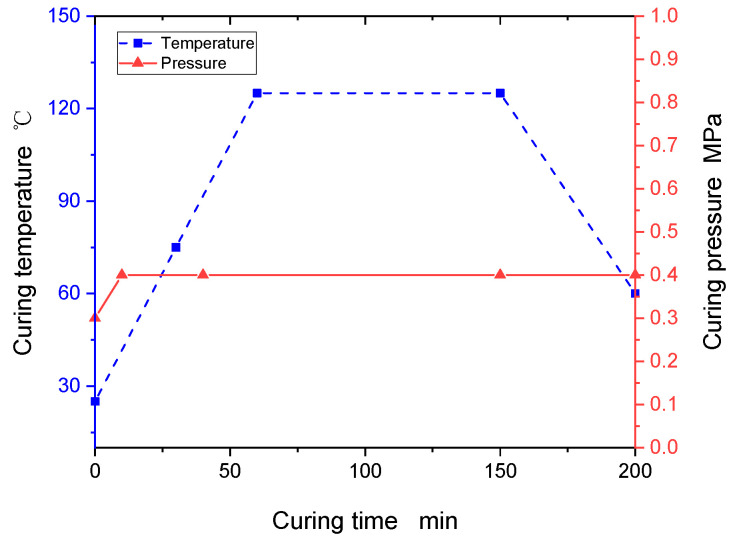
Thermoset composites’ curing process curves.

The thermoset composite samples are laid up and bagged using the classic laminate lay-up and bagging method. During the lay-up process, the boundaries of each prepreg layer in contact with the metal stopper should be strictly laid, the metal stopper is coated with a release agent or release cloth to protect it, and the metal stopper is used on the large plate to provide a straight reference edge to maintain fiber orientation during processing and bonding of the reinforcement sheets. In addition, to ensure the surface accuracy of the specimen, a levelling plate is placed on the bag surface, and a release cloth is spread on the underside of the levelling plate. Thermoset composites are medium temperature curing materials, and the curing curves are shown in Figure 2. To monitor the temperature of the specimens in the hot-pressing tank, two detection thermocouples (T/C) were placed diagonally on each part throughout the curing cycle, and measurements were taken when the hot-pressing tank reached full pressure, with a 10 min interval between the temperature measurements of each thermocouple.

**Figure 3:**
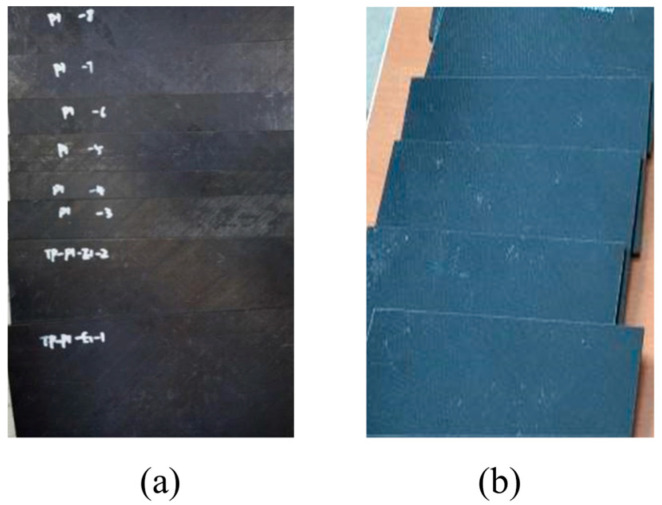
Composite impact specimens and equipment: (**a**) Specimens of TP composite; (**b**) Specimens of TS composite.

The present specimens are two kinds of fiber stacking sequences, which are seen in Table 4. The specimens of TP and TS composite laminates are shown in Figure 3a,b.

### 2.3. Experimental Procedure

**Figure 4:**
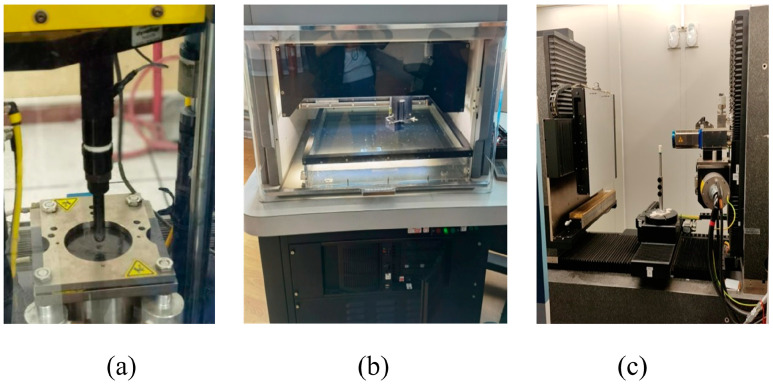
Test equipment and inspection equipment: (**a**) Drop weight impact test bench; (**b**) Ultrasonic testing system; (**c**) X-ray testing device.

All test devices are shown in Figure 4. According to ASTM D7136 [41], the drop-weigh impact test is carried out by using an electric mechanism to lift the impactor. During the impact, the specimen is supported by a base plate with a circular hole in the center and immobilized with clamps. A hemispherical steel impactor with a diameter of 12.7 mm is used for the impact test. The total mass of the punch and counterweight is 7.26 kg. The impact energy includes three levels: E_1_ = 6.67 J/mm, E_2_ = 5.56 J/mm, and E_3_ = 4.45 J/mm. The specimens are measured by an indentation depth gauge when placed for 48 h after impact. Firstly, the overall damage range of the specimen is determined using a C-scan. Then, based on the results of the C-scan analysis, CT technology is used to analyze the damage types and damage mechanisms, and the effects of stacking sequence and material properties on impact damage are discussed. All of the equipment used in the test is shown in Table 5.

## 3. Impact Damage of Composite Laminates

### 3.1. Influence of Materials and Layers on Damage Morphology

**Figure 5:**
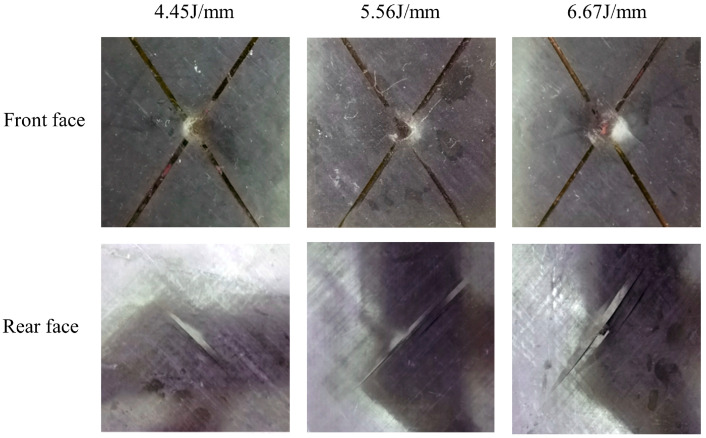
Impact damage of TP composite.

**Figure 6:**
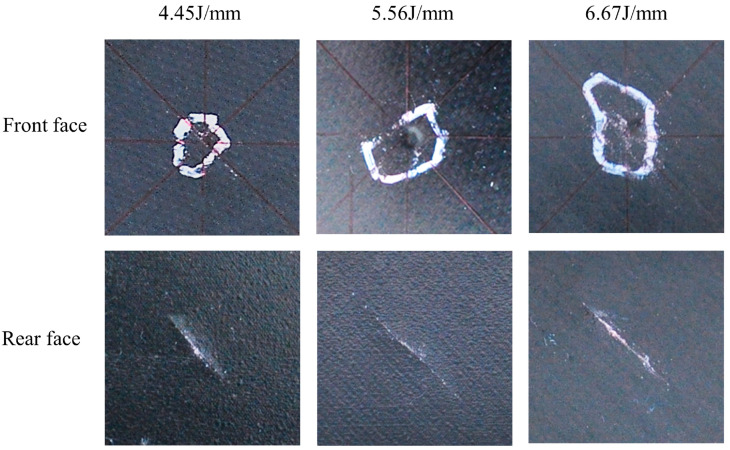
Impact damage of TS composite.

For TP and TS composites, the damage morphology after impact is shown in Figure 5 and Figure 6. It is concluded that the damage that forms on the front surface and the back surface are similar, including the fiber fracture, matrix cracks, and delamination. There is clearly an impact bulge on the back surface and an impact dent on the front surface of specimens. The main reason for the damage is the tension stress and shear stress caused by the bending deformation of the laminate, especially in the lowest plies of the laminate. The tensile stress is perpendicular to the fiber direction in the lowest layer, and is completely transmitted by the matrix, which leads to matrix cracking, the separation between fiber bundles, and the phenomenon of matrix fracture, fiber fracture, and large-scale delamination parallel to the fiber direction.

Under an impact energy of 4.45 J/mm, a small indentation appears on the impact surface of both TP and TS composite materials. This is due to the plastic compression deformation of the matrix after being subjected to the impact. There is no obvious fiber fracture at the indentation of the TP composite plate, and only a slight fiber fracture at the center of the indentation in the TS composite plate. There is a significant matrix cracking phenomenon parallel to the fiber direction on the back surface of the specimen. At an impact energy of 5.56 J/mm, the depth of indentation on the impact surface increases. It can be seen that a few fibers break. The matrix cracking is more serious, showing a strip shape. At a 6.67 J/mm impact energy, the depth of the indentation on the impact surface increases, and a majority of fibers fracture. The matrix cracking phenomenon parallel to the fiber direction on the impact back not only increases, but the fiber fracture phenomenon also occurs.

### 3.2. Influence of Materials and Layers on Impact Force

During impact, the impact force–displacement curve can be used to determine F1 (contact force at the force or slope discontinuity in the force–time curve), the maximum contact force, and is one of the most important parameters used to characterize the mechanical properties of impact. The impact force–displacement curves for thermoplastic and thermoset composites at an impact energy of 6.67 J/mm for different lay-up laminates are shown in Figure 5. The impact force–displacement curves give the change in stiffness of the specimen under impact load (slope of the curve), the maximum displacement, and the damage to the laminate during the impact. The impact process can be roughly divided into three parts: the first part of the curve is linear, for the test specimen is not damaged; the deformation of the test specimen is only in the elastic result, according to the classical laminate theory calculations of the bending stiffness of the P2 ply (ply ratio of 10/80/10), which is significantly lower than that of the P1 ply (ply ratio of 25/50/25), consistent with the graph of the two ply impact force-displacement curve slope and the impact force–displacement curve slope. The slopes and changes of the impact force–displacement curves primarily reflect the flexural stiffness of the laminates. In the second part of the damage emergence stage, the slope declined with the damage area and damage types associated with the damage emergence after the damage began to enter the rapid expansion. The curve was sawtooth until the impact force reached the maximum value; at this time, the energy stored in the laminate has also reached its maximum value. The third stage is the descending stage of the curve; the stored energy of the laminate can cause the punch to rebound, causing a shift in the curve from slow to rapid recovery. The rebound speed of thermoplastics is greater than thermosets.

**Figure 7:**
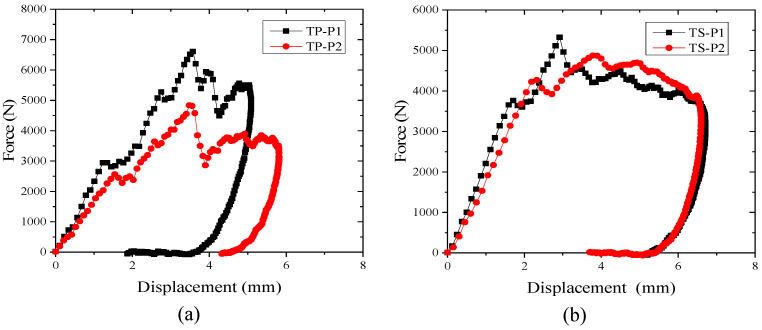
Impact force–displacement curve under 6.67 J/mm. (**a**) TP composite; (**b**) TS composite.

As can be seen from Figure 7a, for thermoset composites TS, when the P1 ply meets about a 3800 N impact force, the plate stiffness begins to change. At this time, the deflection of the plate is about 1.9 mm (predicting that the initial damage of the laminate begins to occur, and at this time, the corresponding impact force is the initial damage valve force). When the initial damage valve force of ply P2 is about 3400 N, the stiffness of the sheet begins to change, and at this time, the deflection of the sheet is about 2.2 mm (predicting that the laminate begins to experience initial damage). The deflection of about 2.2 mm is mainly due to the fact that the contact stiffness and bending stiffness of the P1 laminate is greater than that of the P2 laminate, resulting in a greater impact force when the plate is subjected to an impact. As can be seen from Figure 7b, for the thermoplastic composite TP, the stiffness of the plate starts to change when the P1 layer is impacted at about 2950 N, and the deflection of the plate is about 1.7 mm. For the P2 layer, the stiffness of the plate starts to change when it is impacted at about 2650 N and the deflection of the plate is about 1.85 mm. Comparing the results of the analyses, it can be seen that the initial damage valve force is not only related to the choice of materials of the laminate, but also to the choice of material of the laminate ply, which is the most important factor for the impact of the thermoplastic composites. The initial damage valve force is not only related to the choice of materials for the laminate, but also related to the lay-up order of the laminate. In the same material, the initial valve force of the P2 lay-up with lower stiffness is about 11% lower than that of the P1 lay-up; in the same lay-up order, the initial valve force of the thermoplastic composite is about 28% lower than that of the thermoset composite.

**Figure 8:**
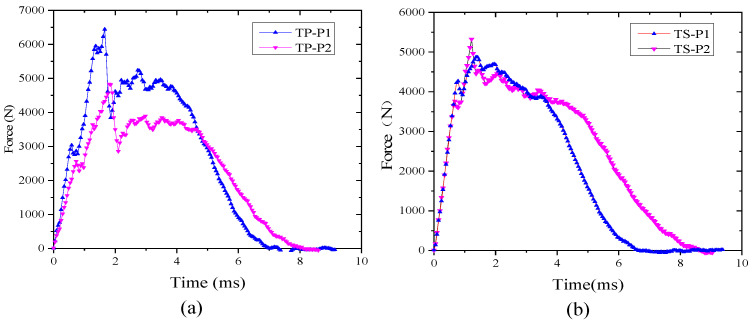
Impact force–time curve under 6.67 J/mm: (**a**) TP composite; (**b**) TS composite.

Figure 8 shows the impact force–time curves of both TP and TS composite laminates at an impact energy of 6.67 J/mm. It can be seen from the results that there is an obvious resonance in the curve of both TP and TS composites. The discontinuity point of slope in the impact force–time curve is the time when the specimens are initially damaged, which can determine the threshold of damage energy (E_1_) and damage force (F1). The initial valve force F1 of the P1 layer is higher than that of the P2 layer, mainly because the P1 layer is thicker than the P2 layer, and the corresponding bending stiffness is also greater than that of the P2 layer. The surface deformation that occurs during the impact process is also greater. From the figure, it can be concluded that whether it is TS composite material or TP composite material, F1 is larger when the laminated plate is thicker. However, as the layer thickness increases, the interaction time between the impact head and the laminate also decreases. This is mainly due to the increase in bending stiffness, which reduces the bending deformation of the laminate during the impact process, resulting in a decrease in the interaction time.

**Figure 9:**
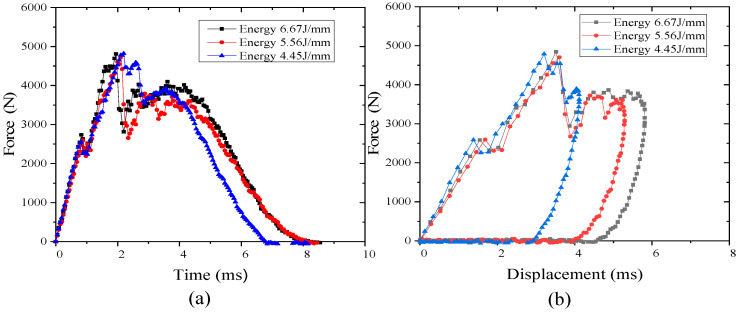
Curve under different impact energies when the TP composite fiber sequence is under the P2 scheme. (**a**) Time–impact force curve; (**b**) Impact force–displacement curve.

Figure 9a shows impact force–time curves under different impact energies (6.67 J/mm, 5.56 J/mm, 4.45 J/mm) when the TP composites are under the P2 scheme. It can be concluded that F1 is basically the same under different impact energies, at about 2650 N. The slope of the impact force–time curve is also basically the same before F1 appears. Figure 9b shows the impact force–displacement curve under different impact energies when the TP composites are under the P2 scheme. With the increase of impact energy, the maximum displacement of the laminate increases continuously, which means the corresponding strain energy increases, and the damage of the laminate becomes more serious.

### 3.3. Influence of Materials and Layers on Energy Absorption

**Figure 10:**
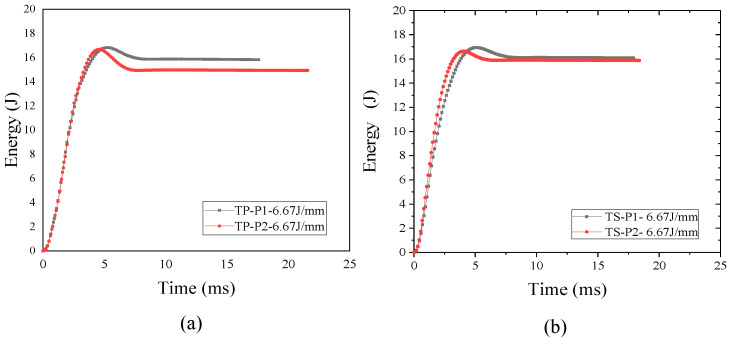
Impact energy–time curve under 6.67 J/mm. (**a**) TP composite; (**b**) TS composite.

**Figure 11:**
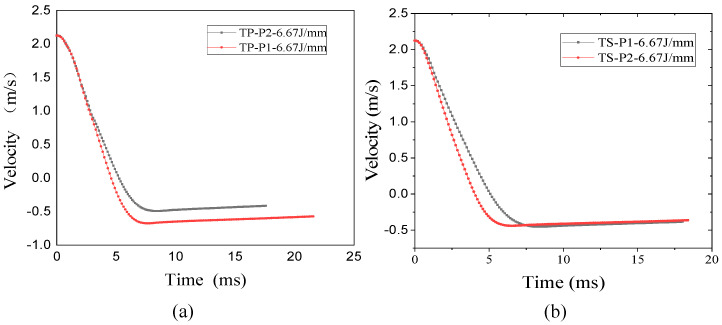
Velocity–time curve under 6.67 J/mm. (**a**) TP composite; (**b**) TS composite.

The change of impact force and speed of composite laminates can reflect its absorption characteristics of energy. Figure 10 shows the impact energy–time curve of TP and TS composites under 6.67 J/mm. For TP composites, the P2 scheme absorbs more energy than P1, which indicates that the lamination scheme of low stiffness with more fiber in the direction of 45° can improve its energy absorption characteristics. For TS composites, the P2 scheme absorbs more energy than P1, but the increase is not as obvious as that of TP composites. Figure 11 shows the velocity-time curves of TP and TS composites under 6.67 J/mm. For all kinds of composites, the impact velocity at the P2 scheme is decreased more than that of P1. But, the reduction of TS composites is not as obvious as that of TP composites. This is because there are more fibers in the 45° direction, causing the stiffness of the plate to be weak. Under the impact loading, fiber fractures and matrix cracks are more likely to occur in weaker laminates, so as to absorb more energy. To sum up, the lamination scheme of lower stiffness can improve its energy absorption characteristics.

### 3.4. Influence of Material and Stacking Sequence on Indentation Depth

After impact, damage to the composite can significantly reduce the residual mechanical properties of the structure. Permanent indentations are left on the frontal impact surface of the specimen. Indentation is therefore one of the most important indicators of damage characterization. Table 6 shows the indentation depth of TP and TS composites after impact at different impact energies. From the data in the table, it can be seen that for the quasi-isotropic sheet P1 and the soft sheet P2, the indentation depth of both the thermoplastic TP and the thermoset TS composites increases with the increase in impact energy; at the same level of impact energy, the indentation depth of sheet P2 is greater than that of sheet P1, and the change in indentation depth of the thermoplastic composites is more obvious. This is due to the fact that sheets with more fibers in the 45° direction are less stiff. Under impact loading, fiber breakage and matrix cracking are more likely to occur in the weaker material, resulting in deeper indentations in the sheet.

**Figure 12:**
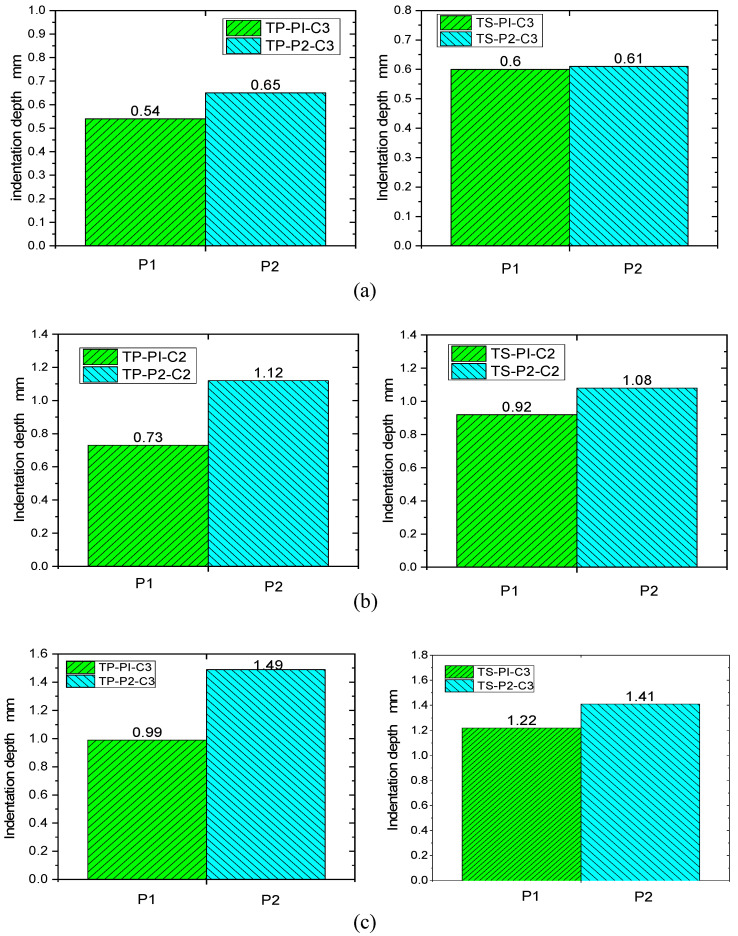
The comparison of indentation depth under different impact energies. (**a**) 4.45 J/mm impact energy; (**b**) 5.56 J/mm impact energy; (**c**) 6.67 J/mm impact energy.

**Figure 13:**
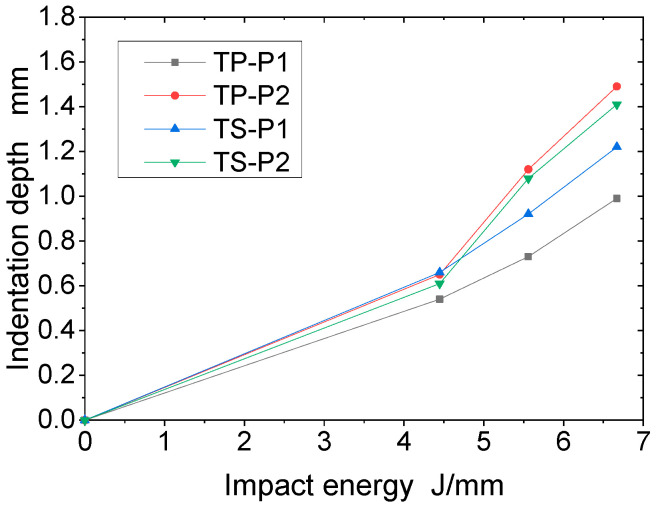
Relation between impact energy and indentation depth.

Figure 12 and Figure 13 show the indentation depth of the thermoplastic composite and thermoset composite for different ply stacking sequences at 4.45 J/mm, 5.56 J/mm, and 6.67 J/mm, respectively. Figure 11 shows the variation of impact energy versus indentation depth for TP and TS for different ply stacking sequences. The indentation depth of the thermoplastic composite is reduced by 10%, 20.7%, and 18.9% for P1 ply compared to the thermoset composite at an impact energy of 4.45 J/mm, 5.56 J/mm, and 6.67 J/mm, respectively, while the damage area of the thermoplastic composite is reduced by 6.6%, 3.7%, and 5.7%, respectively, compared to the thermoset composite for P2 ply. At an impact energy of 4.45 J/mm, the indentation depth of P1 plies is about 20% less than that of P2 plies for thermoset composites, while for thermoplastic composites, the indentation depth of P1 plies is about 2% less than that of P2 plies. The main reason for this is that the P1 ply has a thicker laminate thickness, a higher proportion of 0° plies, and a higher stiffness than the P2 ply, which results in a higher impact force when the punch contacts the laminate and a larger area of delamination damage caused by the higher impact force, while the corresponding indentation depth is smaller.

### 3.5. Influence of Materials and Layers on the Damaged Area

**Figure 14:**
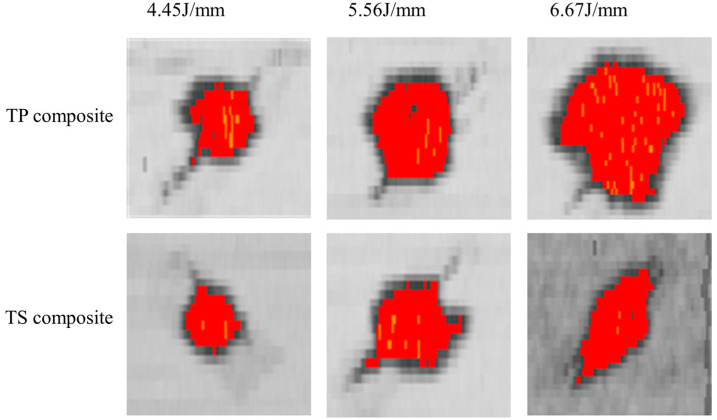
C-scan results of composites at P1 scheme and different impact energy.

**Figure 15:**
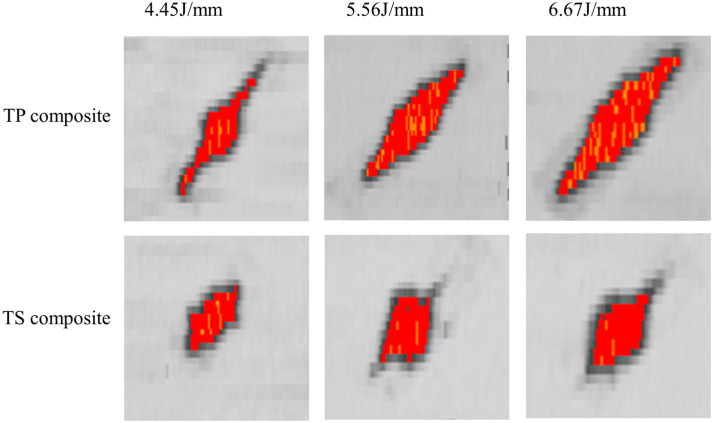
C-scan results of composites at P2 scheme and different impact energy.

The damage images and damage area of the composites were obtained by C-scan ultrasonic testing. The damage images of thermoplastic composites and thermoset composites with P1 and P2 layers after impact at different energy levels are shown in Figure 14 and Figure 15. From the data in the figures and tables, it can be seen that for the quasi-isotropic P1 ply, the delamination damage areas of the thermoplastic composites and thermoset composites are extended in all directions, with a slightly larger area in the 45° direction of the ply, whereas the delamination extension of the ply with a high proportion of soft P2 ply is more pronounced in the 45° direction. This is mainly due to the flexural deformation of the laminate under impact loading, and the tensile loads along and perpendicular to the fiber direction being applied to the impact-backed ply due to the flexural deformation, resulting in fiber bundle separation, interlaminar slippage, and fiber breakage.

**Figure 16:**
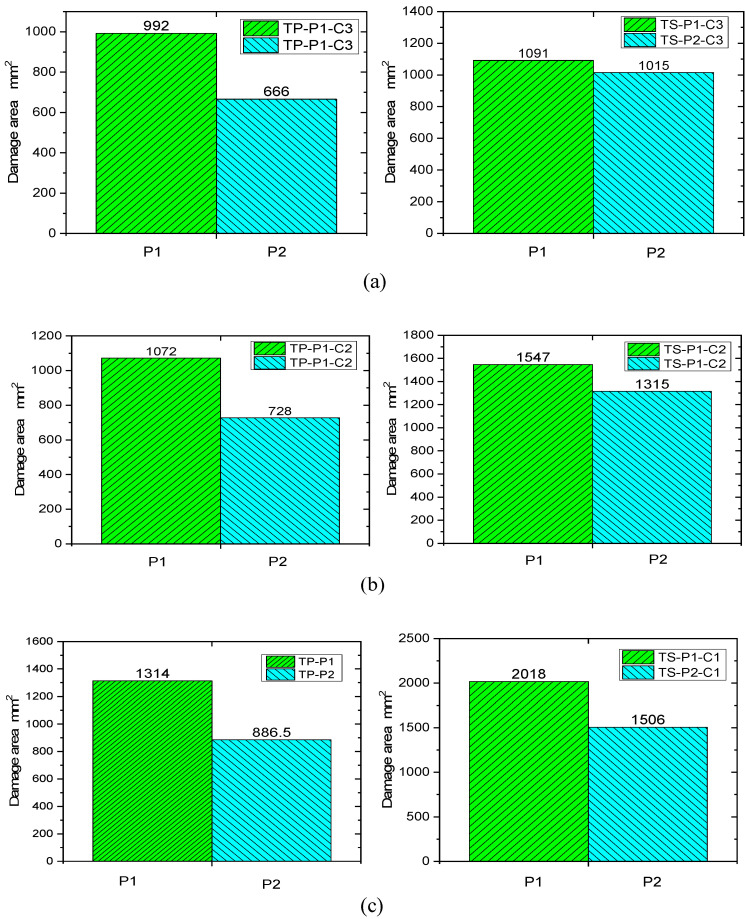
The comparison of damage area at different impact energies. (**a**) 4.45 J/mm impact energy; (**b**) 5.56 J/mm impact energy; (**c**) 6.67 J/mm impact energy.

**Figure 17:**
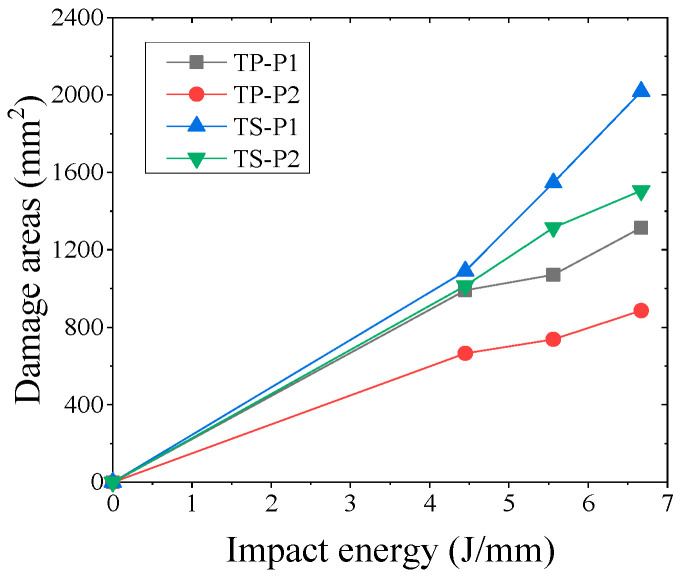
Relation between impact energy and damage area.

A comparison of the damage area of TP and TS composites for different fiber stacking sequences at impact energies of 4.45 J/mm, 5.56 J/mm, and 6.67 J/mm is shown in Figure 16. The variation curves of impact energy and the damage area of TP and TS composites for different fiber stacking sequences are shown in Figure 17. It can be seen from the figure that for TP composites, the damage area of all composites gradually increases with the increase in impact energy. For the impact energies of 4.45 J/mm, 5.56 J/mm, and 6.67 J/mm, the damage areas of the thermoplastic composites compared to the thermoset composites were reduced by 9.1%, 30.7%, and 34.9%, respectively, for the P1 lamination, whereas for the P2 lamination, the damage areas of the thermoplastic composites compared to the thermoset composites were reduced by 34.4%, 43.9%, and 41.4%, respectively. At an impact energy of 4.45 J/mm, the damage area of the P1 ply increased by about 30% compared to the P2 ply for thermoset composites, while the damage area of the P1 ply increased by about 7% compared to the P2 ply for thermoplastic composites. The main reason for this is that the thickness of the P1 composite is greater, the proportion of 0° plies is higher, and the stiffness is also greater; the impact force when the punch contacts the laminate is greater and the delamination damage area caused by the impact force is also greater, and the increase is more obvious for thermoset composites than for thermoplastic composites.

Furthermore, comparing the damage areas and indentation depths in Table 6 and Table 7, it can be seen that for the same impact energy, the damage area of the P1 layer is larger than the delamination damage area of the P2 layer. However, the indentation depth is opposite, and the indentation depth of the P1 ply is smaller than that of the P2 ply.

### 3.6. Internal Mesoscopic

**Figure 18:**
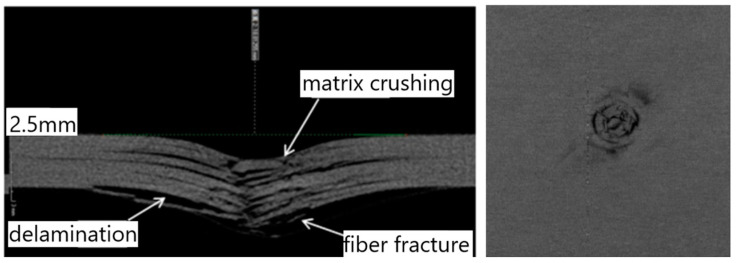
CT image of the specimen under 6.67 J/mm impact energy.

In order to further understand the internal micro-damage of the specimens after impact, the high-resolution CT detection system Y.CT MODULAR is used to conduct non-destructive CT scanning of the specimens after impact, that is, to conduct non-destructive CT scanning of the central section of the material after low speed impact through X-ray technology. Figure 18 shows the CT image of the P1 ply test piece under 6.67 J/mm impact energy. It can be clearly seen from the figure that the material in contact with the impact head is impacted to form an arc-shaped pit. The back of the test piece is bent and bulged, and the material in contact with the impact head has the phenomenon of matrix crushing. Under the impact load, there is obvious fiber fracture in the contact area of the punch, but there is more interlaminar delamination damage and fiber fracture at the impact back, and the delamination damage extends beyond the pit along the plane.

**Figure 19:**
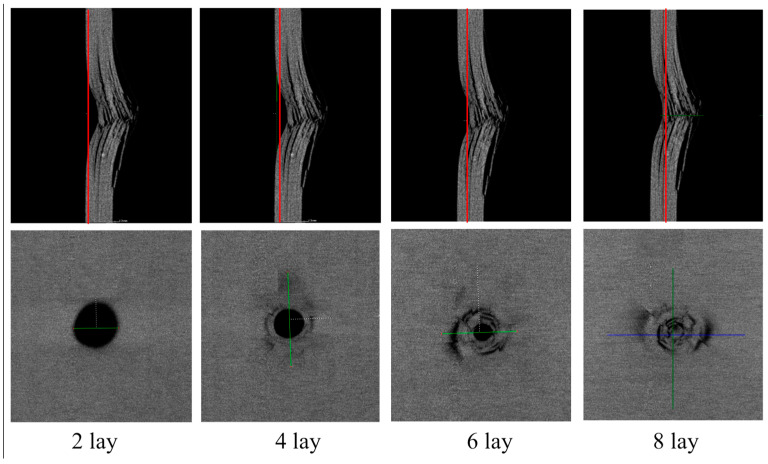

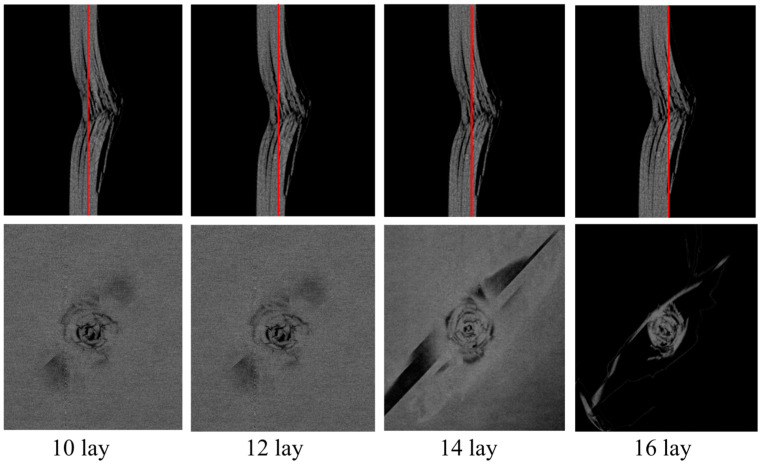
CT images of damage to each layer of the specimen under 6.67 J/mm impact energy.

By slicing the 3D view along the thickness direction of the laminate, the damaged details of each layer of the laminate can be obtained. Figure 19 shows the CT images of the damage of each layer of the P2 layer test piece under 6.67 J/mm impact energy. From the figure, it can be seen that the industrial CT system is the best method for detecting low-velocity impact damage in composite laminated structures. Compared to the projection of all layer damage structures obtained by C-scan, the industrial CT system can clearly see the damage morphology and size of each layer from each profile, and can accurately measure the depth of the damage indentation. Based on the detection results of each layer, 3D damage area reconstruction can be performed very well. 

**Figure 20:**
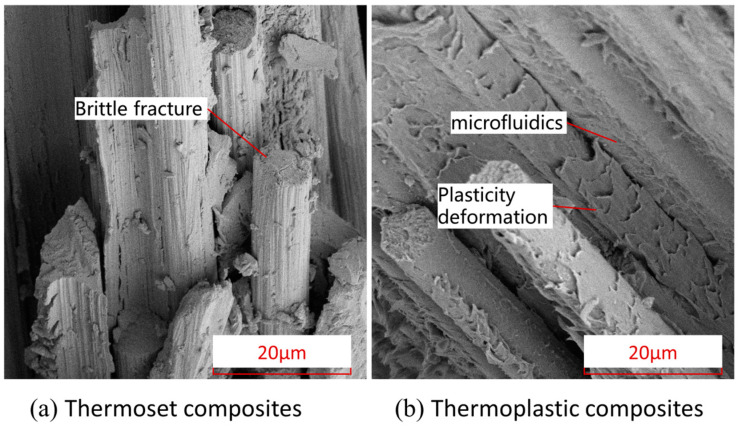
The SEM images of the thermoset composite TS and thermoplastic composite TP damaged at the fiber–matrix interface.

In order to further understand the fiber damage, matrix damage, and interfacial damage of thermoset and thermoplastic composites in the damage region under low speed impact, and to analyze the microscopic differences in damage mechanisms between thermoplastic and thermoset composites, the Apreo scanning electron microscope from Thermo Fisher Scientific (Thermo Scientific, Waltham, MA, USA) was used to compare and analyze the damaged region of thermoplastic and thermoset composites after impact. The cross sections of the damaged areas of thermoplastic and thermoset composites after impact were compared and analyzed. The SEM images of the thermoset (TS) and thermoplastic (TP) composites damaged at the fiber–matrix interface are shown in Figure 20. As can be seen from the figure, compared with the thermoset composites, the thermoplastic composite has obvious plastic deformation of the matrix at the fiber–matrix interface junction, and the shedding of the fiber–resin interface is relatively small. There is plastic deformation of the microfluidic phenomenon in the TP matrix, which shows obvious ductile fracture characteristics, while most of the fracture areas of the TS matrix have edge flake fracture, which is an obvious brittle fracture characteristic. It is this toughness characteristic of thermoplastic composites that gives them good impact resistance properties.

## 4. Simulation Analysis

The damage constitutive relationship, damage initiation criteria, damage propagation, elastic–plastic shear failure criterion, and delamination failure criteria of CF/PEEK composite materials in this article are consistent with a previous article published by the author. In addition, the reliability of the analysis results has also been validated in the references [42].

**Figure 21:**
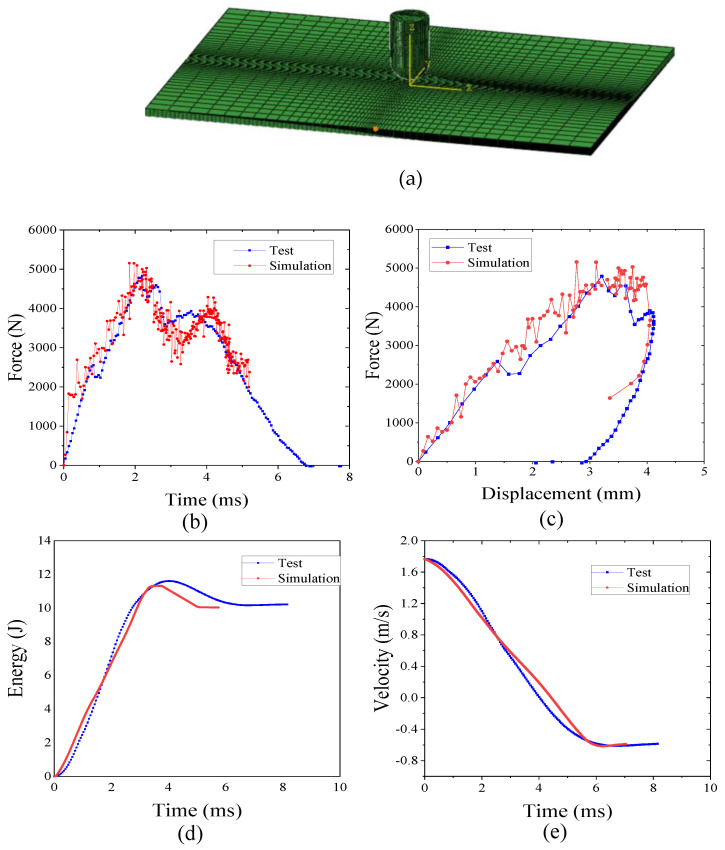
Comparison of simulation and tests of P2 laminated TP composites at 4.45 J/mm impact. (**a**) Simulation model; (**b**) Impact force–time curve; (**c**) Impact force–displacement curve; (**d**) Impact energy–time curve; (**e**) Velocity–time curve.

The three-dimensional finite element model of CF/PEEK composite with the P2 scheme is established to simulate the process of impact damage. By using the commercial finite element software ABAQUS 6.14-1, the composite laminate utilizes the C3D8R unit, and the COH3D8 unit is adopted for interlamination. The impact head is a 12.7 mm hemispherical structure, and the impact energy is 4.45 J/mm, using the C3D10M unit. The tangential contact between the impact head and the laminate is defined as penalty contact, and the friction coefficient is 0.15. The normal contact is set as a ‘hard’ contact. The mesh of the model is shown in Figure 21a. In order to predict the failure mechanism of the present TP composites, we adopt a progressive damage model based on the Hashin failure criteria, and it is implemented in ABAQUS/Explicit by embedding user subroutine VUMAT, which has been developed in our previous works [42,43,44].

Figure 21a,b show a comparison of the simulation test results of impact force–time and impact force–displacement during the impact process. As can be seen from the figure, at about 0.5 ms, the displacement is 1.51 mm, the impact force is 2650 N, the curve oscillation starts to be violent, and the slope of the curve seems to decrease for the first time, which indicates that the laminate starts to appear damaged. At this time, the impact force is the valve force for the initial occurrence of damage to the laminate; the impact force reaches the maximum value at about 2 ms, the maximum impact force of the experimental test is 4538 N, and the corresponding displacement is 3.537 mm. The numerical analysis results after filtering the maximum impact force is 4736 N, the corresponding displacement is 3.446 mm, and the error of the maximum impact force is 4.36%, which shows that the calculation model has good reliability; in the contact force after the maximum, the punch began to rebound, and in the rebound stage of the numerical analysis of the contact force, the test results are slightly larger and the decline rate is faster. This may be due to the fact that the damage variable is directly deleted after reaching one in the numerical calculation.

Figure 21c,d show a comparison of the simulation test results of the impact energy–time and velocity–time curves during the impact process. As can be seen from the figures, when the impact head hits the thermoplastic composite laminate, the initial kinetic energy of the impact head begins to be transferred to the laminate. Initially, the laminate absorbs the kinetic energy of the impact head through its elastic deformation, and after the initial damage to the laminate occurs, more energy is dissipated due to the cracking of the interlaminar adhesive layer of the laminate, the fracture of the fibers and matrix in the face, and the crack propagation in the adjacent plies. In the impact process, the energy absorption becomes larger and larger as the speed gradually decreases. The kinetic energy of the impact head also gradually decreases; when the speed of the impact head is zero, the kinetic energy of the impact head is all transferred to the laminate, and the stored elasticity of the laminate can then be transferred to the impact head in the opposite direction, causing the impact head to rebound.

**Figure 22:**
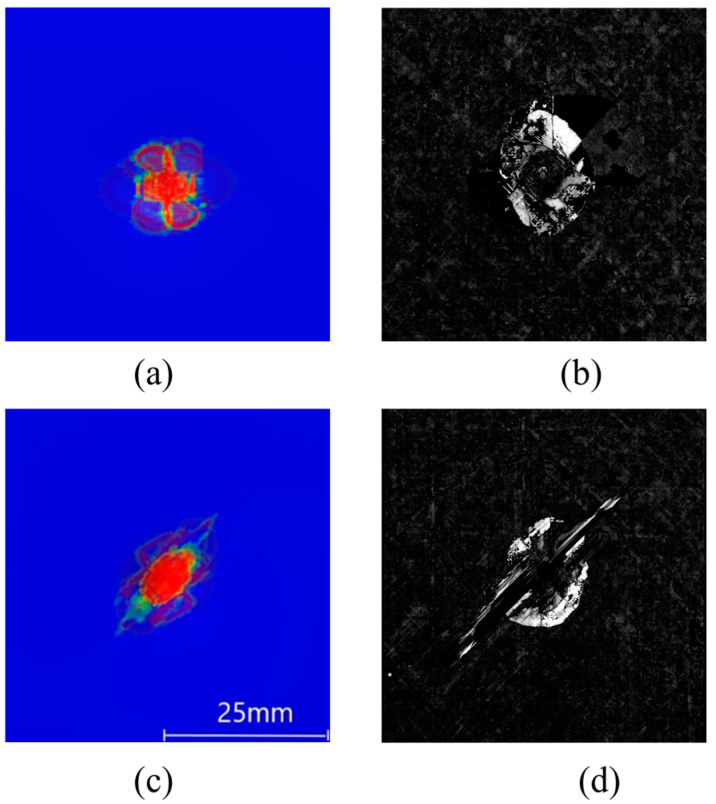
Delamination comparison of simulation and tests of TP composites at a 4.45 J/mm impact. (**a**) P1 layer simulation result; (**b**) P1 layer C-scan result; (**c**) P2 layer simulation result; (**d**) P2 layer C-scan result.

In order to better understand the internal delamination and matrix cracking damage of the test piece after impact, the damaged area of the test piece after impact is scanned by C-scan, and the scanning results are compared with the damage SDEG of the adhesive layer unit obtained by numerical analysis. Since the C-scan results are the superposition of the damage area projections of all layers in the actual impact, the superposition results of all adhesive layer units are selected when taking the finite element results. Figure 22 shows the delamination comparison of simulation and tests of TP composites at a 4.45 J/mm impact. Figure 22a,b show the delamination comparison of the simulation result and C-scan result for the P1 scheme. Figure 22c,d show the delamination comparison of the simulation result and C-scan result for the P2 scheme. It can be seen that the calculation model is relatively accurate in simulating the damage of matrix cracking and delamination.

**Figure 23:**
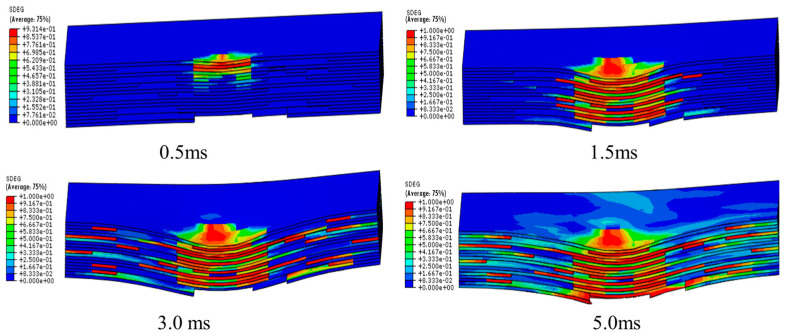
SDEG of the cohesive layer at different times.

In the process of low velocity impact, the main damage forms are matrix cracks, delamination, fiber fractures, etc. Matrix cracks and interlamination damage occur first. Generally, composite laminates appear as matrix damage first at 2 J–4 J. Figure 23 shows the stiffness degradation (SDEG) of the interlamination at different times. The initial damage in the interlamination occurs at 0.5 ms. The damage is mainly located on the upper surface in contact with the impact head. As the impact head moves down, the laminate is punched out of the pit. The lower surface of the laminate in the impacted area is severely strained, so the interlamination cracks seriously on the lower surface.

**Figure 24:**
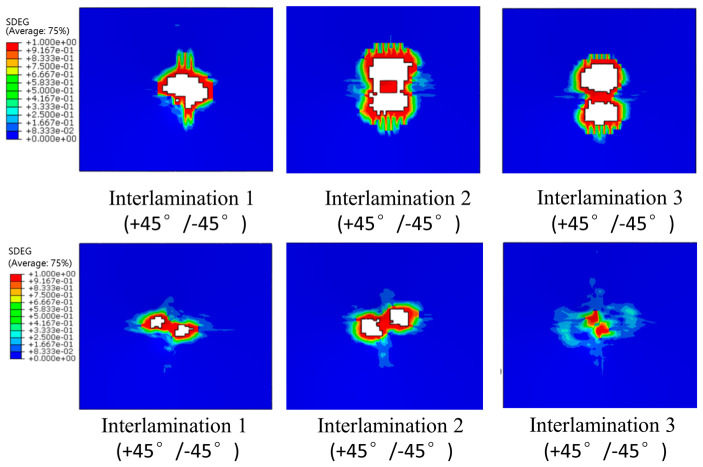
Interlamination SDEG at different interface.

In the P2 scheme, interlamination 1 is closest to the lower surface, and interlamination 19 is closest to the upper surface (impact surface). Figure 24 shows the SDEG under an impact energy of 4.45 J/mm. It can be seen from the figure that the delaminated area near the lower surface is relatively large. On the contrary, the damaged area near the impact surface is relatively small. This is mainly because the bend deformation is larger in the lower surface, thus the bending stress is serious and the delaminated area becomes larger. On the one hand, the matrix cracks expand faster in the fiber direction than perpendicular to the fiber direction. On the other hand, the cracks will expand rapidly along the thickness direction. Furthermore, if the fiber direction of the adjacent layer is in a different orientation, the matrix crack will be blocked, and it will expand in interlamination to form delaminations.

**Figure 25:**
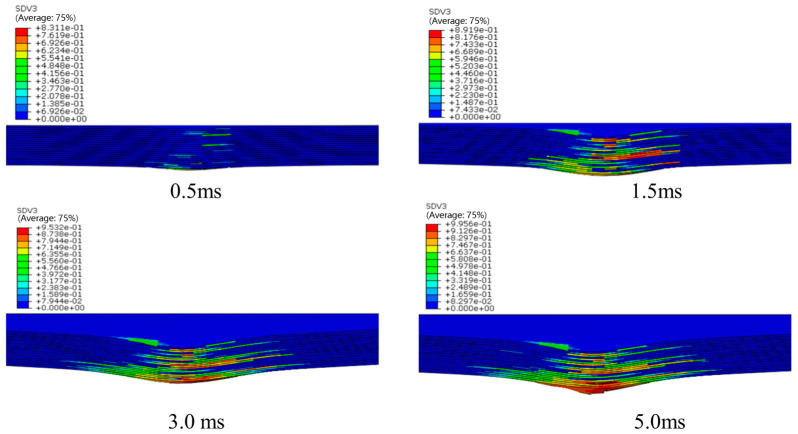
Matrix tensile damage at different times.

Figure 25 shows the matrix tensile damage at a 4.45 J/mm impact energy. Bending deformation occurs in the impact area, and the lower surface of the lamination bears the tensile load. When *t* = 0.5 ms, matrix tensile damage occurs. As the impact load increases sharply in a short time, the matrix tensile damage also occurs on the edge of the indentation on the impact surface. With the increasing impact load, the tensile load of the matrix on the lower surface expands both in the plane and in the thickness direction.

## 5. Conclusions

In this paper, the impact tests of TP and TS composites have been carried out. In addition, the displacement–time curves, force–time curves, velocity–time curves, and displacement–force curves under different impact energies have been obtained. The characteristics of different kinds of composites, such as stiffness change, contact force, damage absorption, and energy absorption, have been studied. In addition, the damaged area of the composites has been measured by an ultrasonic testing system. Finally, the damage growth process of composite materials is simulated by the finite element method. The main conclusions are as follows:

(1) Through the comparative analysis of the initial damage valve force of the low-speed impact of different materials with different lay-ups, it is found that the initial damage valve force is not only related to the choice of laminate materials, but also to the lay-up order of the laminate. For the same material, the initial valve force of ply P2 with a lower stiffness is about 11% lower than that of ply P1; for the same ply sequence, the initial valve force of thermoplastic composite TP is about 28% lower than that of thermoset composite. For the same type of ply and the same type of material, under different impact energies, the initial damage valve force F1 of thermoset composites and thermoplastic composites is basically the same, and the slopes of the impact force–time curves are also basically the same (i.e. contact stiffness) before the appearance of F1, indicating that the magnitude of the initial valve force is mainly related to the contact stiffness of the laminate.
(2) For quasi-isotropic sheet P1 and soft sheet P2, the indentation depth of both thermoplastic material and thermoset composite material increases with the increase of impact energy; at the same level of impact energy, the indentation depth of sheet P2 is larger than that of sheet P1, and the change of indentation depth of thermoplastic composite material is more obvious. This is because the sheet with more fibers in the 45° direction is less rigid. Under impact loading, fiber breakage and matrix cracking are more likely to occur in the weaker material, resulting in greater indentation depths in the laminate.
(3) For the quasi-isotropic ply P1, the delamination damage regions of the thermoplastic composites and thermoset composites are all extended, slightly larger in the 45° ply direction, whereas the soft ply P2, which contains a high proportion of 45°, has a more pronounced delamination extension in the 45° direction. This is mainly due to the bending deformation of the laminate under the impact load, and the impact back ply is subjected to tensile loads along and perpendicular to the fiber direction due to the bending deformation, resulting in fiber bundle separation, interlaminar slippage, and fiber breakage.
(4) Comparing the scanning electron microscope SEM results of thermoplastic composites and thermoset composites, it was found that, compared with thermoset composites, the thermoplastic composite had significant matrix plastic deformation at the fiber/matrix interface junction, and relatively small fiber-resin interface shedding. There was also a microfluidic phenomenon of plastic deformation in the TP matrix that showed obvious ductile fracture characteristics, while most of the fracture regions of the TS matrix were edge flake fractures; brittle fracture characteristics are obvious. It is this toughness characteristic of thermoplastic composites that gives them good impact resistance.
(5) In addition, the established damage model based on continuous damage mechanics (CDM) can effectively predict the various damage modes of thermoplastic composites during low-speed impact. Comparing the analytical results with the experimental results, the results show that the error is 5.26% in the simulation test of the initial damage threshold force, with an impact energy of 4.45 J/mm. The error of the maximum impact force is 4.36%. The simulated impact energy and impact velocity curves are in good agreement with the experimental results, indicating that the damage model has good reliability.

This paper only analyses the damage characteristics and damage mechanism of thermoplastic and thermoset composites under low-velocity impacts at impact energies of 4.45 J/mm, 5.56 J/mm, and 6.67 J/mm, and more damage analyses under impact energies can be added later, including re-studies of the post-impact residual compressive strength and fatigue growth characteristics of the damaged region after impact, in order to provide technical reserves for the design of damage tolerance for thermoplastic composite structures.

## Figures and Tables

**Table 1:** Typical mechanical properties of carbon fibers [40].

	Tension Modulus (GPa)	Ultimate Tension Strength (MPa)	Elongation (%)
AS4D	231	4347	1.88
CCF300	230	4210	1.78

**Table 2:** Typical mechanical properties of matrix [40].

	Tension Modulus (MPa)	Tension Strength (MPa)	Elongation (%)	Fracture Toughness (J/mm)
PEEK	3.8	94	50	2000
Epoxy	3.58	105	1.9	420

**Table 3:** Mechanical parameters of AS-4D/PEEK TP and CCF300/Epoxy composites [40].

	Density (MPa)	Poisson’s Ratio	Elastic Modulus (GPa)		Share Modulus (GPa)	Tensile Strength (MPa)		Compression Strength (MPa)		Shear Strength (MPa)
	ρ	*v*	E_1_	E_2_	G_12_	*σ_t_*_1_	*σ_t_*_2_	*σ_c_*_1_	*σ_c_*_2_	*τ*_12_
AS-4D/PEEK	1580	0.3	130	9.7	5.2	2280	69	1300	208	152
CCF300/Epoxy	1600	0.3	130	9.3	4.1	1673	68	1436	257	136

**Table 4:** Lamination scheme in the impact test.

	Mark	Fiber Stacking Sequence
TP composite	P1	[45°/0°/−45°/90°]_3S_
	P2	[45°/−45°/90°/45°/−45°/45°/−45°/0°/45°/−45°]s
TS composite	P1	[45°/0°/−45°/90°]_3S_
	P2	[45°/−45°/90°/45°/−45°/45°/−45°/0°/45°/−45°]s

**Table 5:** List of test equipment.

Equipment	Model	Precision
Drop hammer impact test bench (INSTRON, Boston, MA, USA)	9250HV	±0.5%
Depth gauge (KENTA, Singapore)	KT5-231-66	0.01 mm
Vernier caliper (MITUTOYO, Kawasaki City, Japan)	—	±0.02 mm
Ultrasonic testing system (Sonoscan, IL, USA)	IUCS—II	—
X-ray testing device (YXLON, Hamburg, Germany)	Y.CT Modular	1%

**Table 6:** Indentation depth of composite materials.

Material	Layer	Number	Energy of Impact		Indentation Depth (mm)		CV%
			J/mm	J	Single Value	Average Value	
TP composite	P1	TPP1C1-01	6.67	20.01	1.11	0.99	9.59
		TPP1C1-02	6.67	20.01	0.97		
		TPP1C1-03	6.67	20.01	0.88		
		TPP1C2-04	5.56	16.68	0.67	0.73	10.35
		TPP1C2-05	5.56	16.68	0.84		
		TPP1C2-06	5.56	16.68	0.69		
		TPP1C3-07	4.45	13.35	0.54	0.54	12.94
		TPP1C3-08	4.45	13.35	0.45		
		TPP1C3-09	4.45	13.35	0.62		
	P2	TPP2C1-01	6.67	16.68	1.40	1.49	8.07
		TPP2C1-02	6.67	16.68	1.41		
		TPP2C1-03	6.67	16.68	1.66		
		TPP2C2-04	5.56	13.90	1.12	1.12	11.99
		TPP2C2-05	5.56	13.90	1.29		
		TPP2C2-06	5.56	13.90	0.96		
		TPP2C3-07	4.45	11.13	0.68	0.65	12.37
		TPP2C3-08	4.45	11.13	0.54		
		TPP2C3-09	4.45	11.13	0.73		
TS composite	P1	TSP1C1-01	6.67	20.01	1.38	1.22	10.39
		TSP1C1-02	6.67	20.01	1.07		
		TSP1C1-03	6.67	20.01	1.21		
		TSP1C2-04	5.56	16.68	1.05	0.92	10.06
		TSP1C2-05	5.56	16.68	0.89		
		TSP1C2-06	5.56	16.68	0.83		
		TPP2C3-07	4.45	11.13	0.68	0.60	13.56
		TPP2C3-08	4.45	11.13	0.49		
		TPP2C3-09	4.45	11.13	0.64		
	P2	TSP2C1-01	6.67	16.68	1.27	1.41	8.13
		TSP2C1-02	6.67	16.68	1.40		
		TSP2C1-03	6.67	16.68	1.55		
		TSP2C2-04	5.56	13.90	1.08	1.08	9.42
		TSP2C2-05	5.56	13.90	1.21		
		TSP2C2-06	5.56	13.90	0.96		
		TSP2C3-07	4.45	11.13	0.63	0.61	11.48
		TSP2C3-08	4.45	11.13	0.52		
		TSP2C3-09	4.45	11.13	0.69		

**Table 7:** Damage area of composite materials.

Material	Layer	Number	Energy of Impact		Damage Area (mm^2^)		CV%
			J/mm	J	Single Value	Average Value	
TP composite	P1	TPP1C1-01	6.67	20.01	1332	1314	11.26
		TPP1C1-02	6.67	20.01	1124		
		TPP1C1-03	6.67	20.01	1485		
		TPP1C2-04	5.56	16.68	979	1072	9.79
		TPP1C2-05	5.56	16.68	1019		
		TPP1C2-06	5.56	16.68	1219		
		TPP1C3-07	4.45	13.35	1164	992	12.34
		TPP1C3-08	4.45	13.35	926		
		TPP1C3-09	4.45	13.35	887		
	P2	TPP2C1-01	6.67	16.68	913	887	10.84
		TPP2C1-02	6.67	16.68	989		
		TPP2C1-03	6.67	16.68	758		
		TPP2C2-04	5.56	13.90	766	738	8.93
		TPP2C2-05	5.56	13.90	647		
		TPP2C2-06	5.56	13.90	801		
		TPP2C3-07	4.45	11.13	648	666	10.54
		TPP2C3-08	4.45	11.13	760		
		TPP2C3-09	4.45	11.13	591		
TS composite	P1	TSP1C1-01	6.67	20.01	2015	2018	11.97
		TSP1C1-02	6.67	20.01	2316		
		TSP1C1-03	6.67	20.01	1724		
		TSP1C2-04	5.56	16.68	1521	1547	11.62
		TSP1C2-05	5.56	16.68	1341		
		TSP1C2-06	5.56	16.68	1779		
		TSP1C3-07	4.45	13.35	1253	1091	12.12
		TSP1C3-08	4.45	13.35	1091		
		TSP1C3-09	4.45	13.35	929		
	P2	TSP2C1-01	6.67	16.68	1527	1506	10.24
		TSP2C1-02	6.67	16.68	1683		
		TSP2C1-03	6.67	16.68	1307		
		TSP2C2-04	5.56	13.90	1375	1315	16.83
		TSP2C2-05	5.56	13.90	1019		
		TSP2C2-06	5.56	13.90	1551		
		TSP2C3-07	4.45	11.13	1024	1015	9.99
		TSP2C3-08	4.45	11.13	1135		
		TSP2C3-09	4.45	11.13	887		

## Data Availability

Data are contained within the article.
